# Supplementary figures and images for: Strain- and plasmid-level deconvolution of a synthetic metagenome by sequencing proximity ligation products
Source: PeerJ. 2014 May 27;2:e415. doi: 10.7717/peerj.415 (PMC4045339; doi:10.7717/peerj.415)

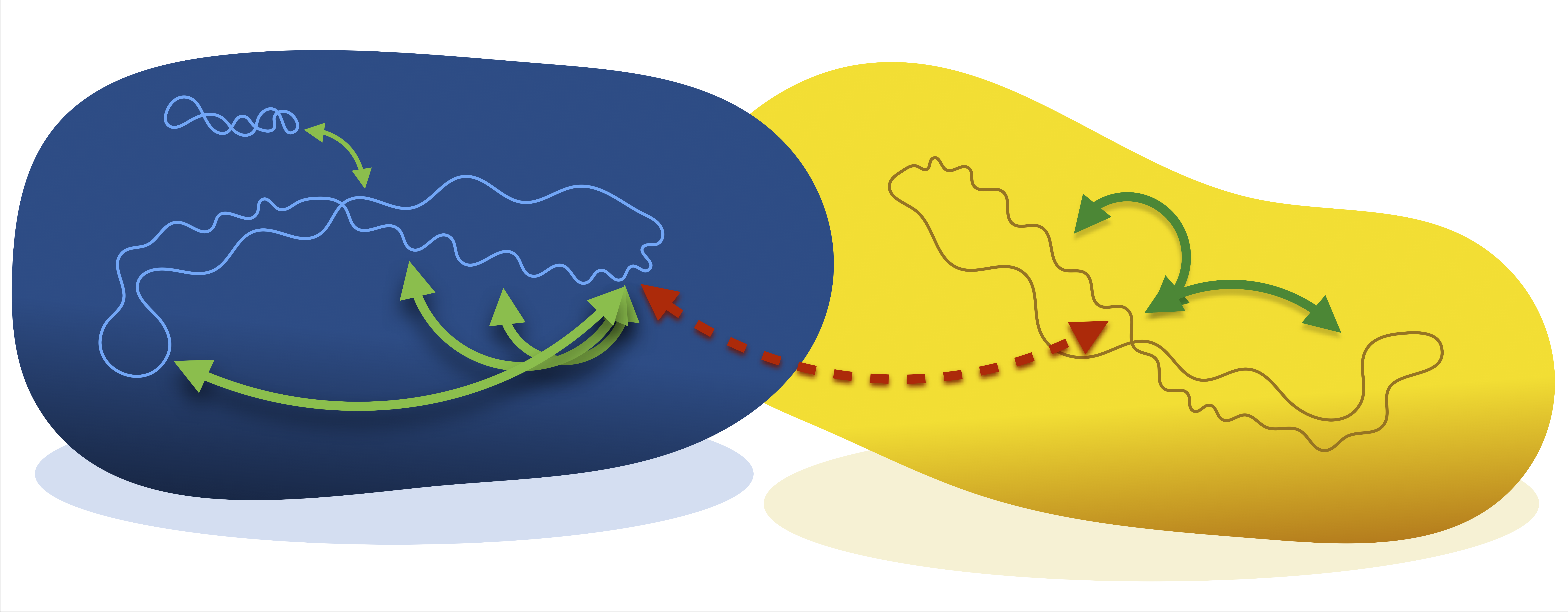

Supplement: Figure S1 — Two bacterial cells are illustrated, each containing a single circular chromosome. For one genomic region in each of the two species, examples of associations that are likely (green; red is “not likely”) to be derived from Hi-C are illustrated. [file peerj-02-415-s001.png]

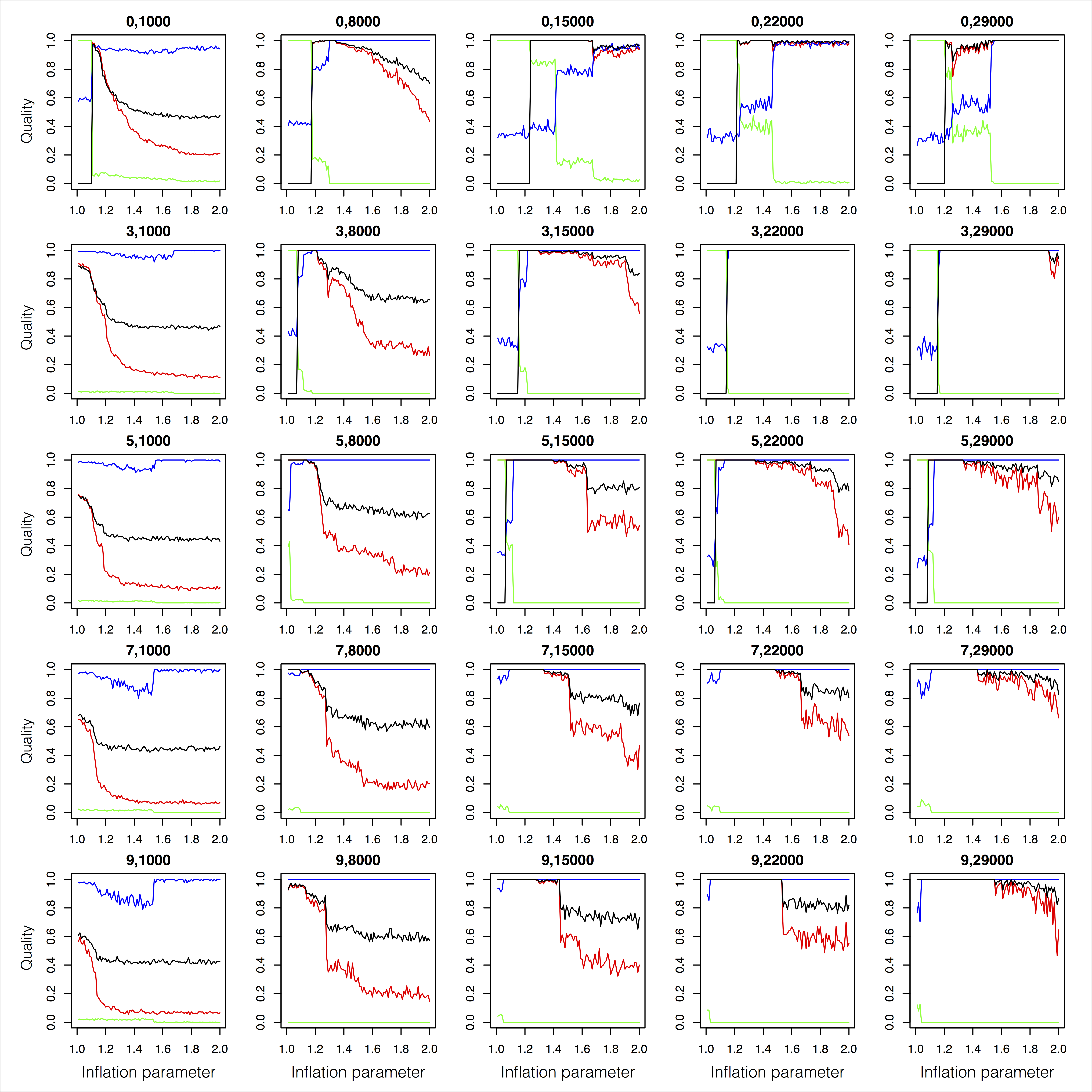

Supplement: Figure S2 — A small-multiples plot is showing 5 × 5 combinations of contact minimum (top to bottom; 0, 3, 5, 7, 9) and contig size minimum (left to right; 1,000, 8,000, 15,000, 22,000, 29,000) thresholds. For each parameter combination, line plots show the quality (y-axis) of clustering solutions performed for inflation values in the interval [1, 2]. The quality of clustering solutions is measured in terms their true-positive rate (red), false-positive rate (green), positive predictive value (blue), and negative predictive value (black) are shown. [file peerj-02-415-s002.png]

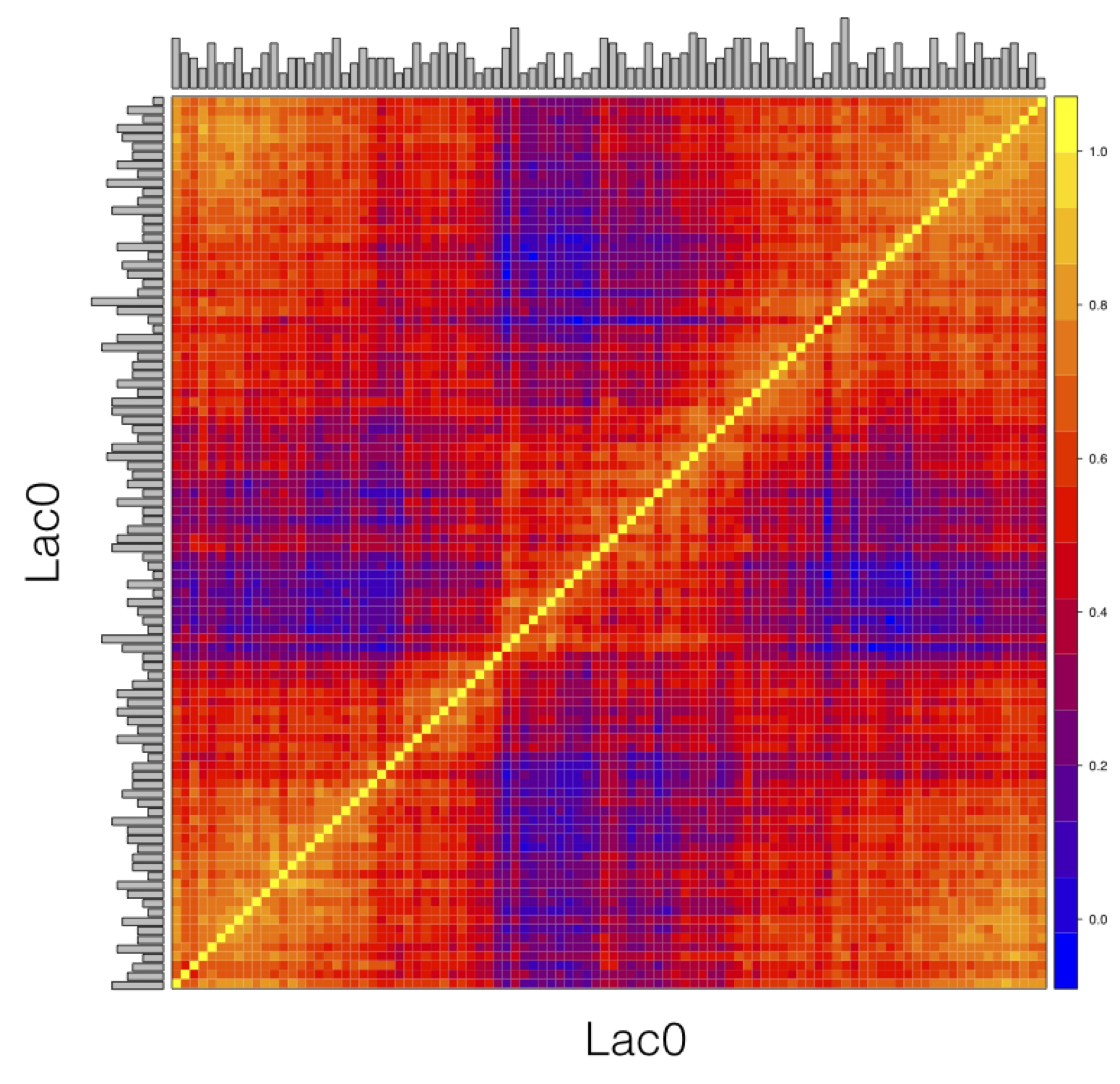

Supplement: Figure S3 — Contact frequency is visualized as a heat map, after normalization and application of the spearman rank correlation (matrix elements are the spearman correlation of the row and column of which they are the intersection). Circularity is apparent in the elevated contact between either end of the reference assembly sequence. [file peerj-02-415-s003.png]

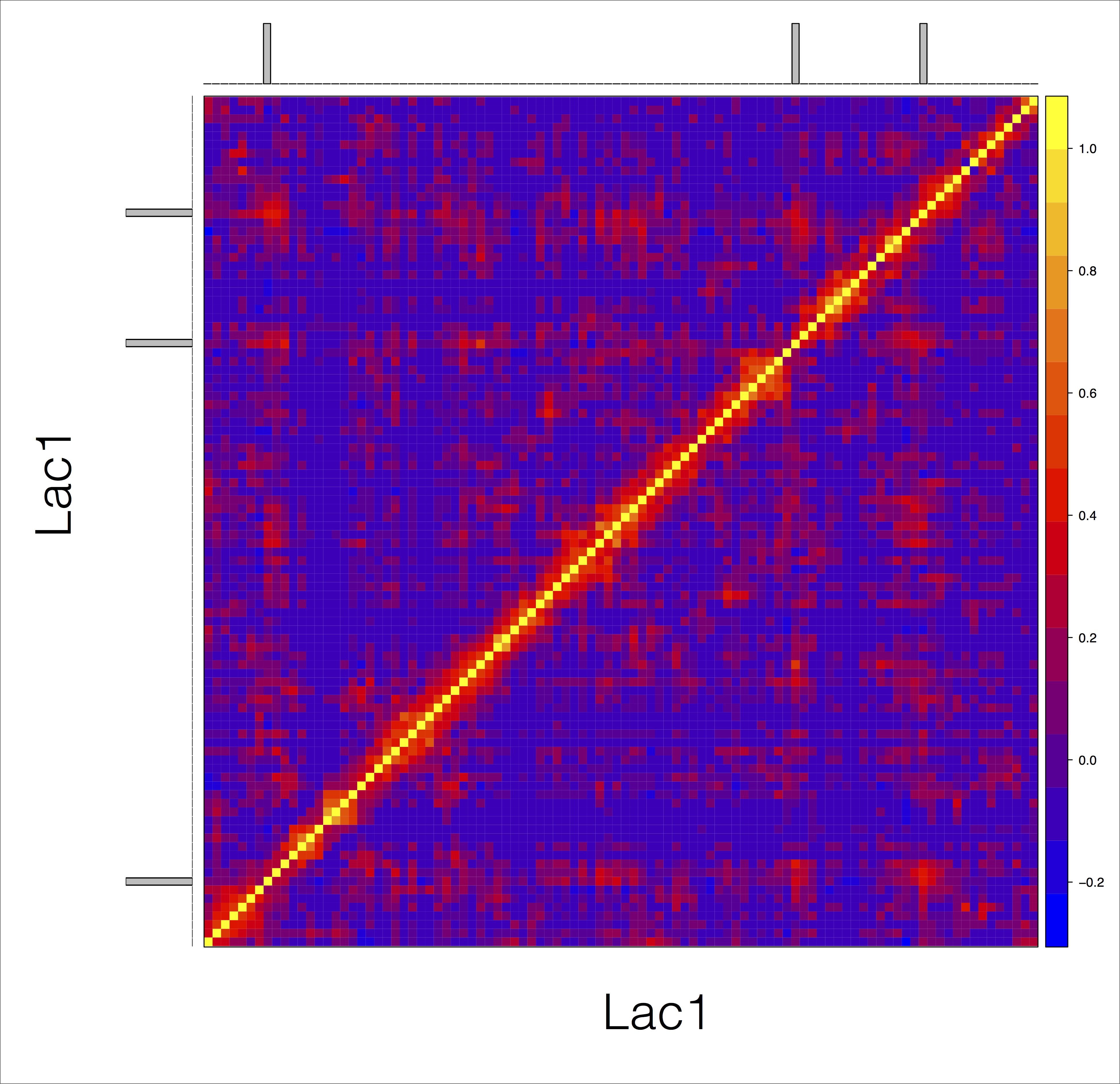

Supplement: Figure S4 — Contact maps show the number of Hi-C read pairs associating each region of the L. brevis plasmid 1. Contact values are Spearman rank correlation transformed following normalization. Pixels are sized to represent interactions between blocks sized at 1% of the interacting sequence. A minimal signal of circularity is apparent with enrichment for contact between the minimum and maximum positions within the reference assembly. [file peerj-02-415-s004.png]

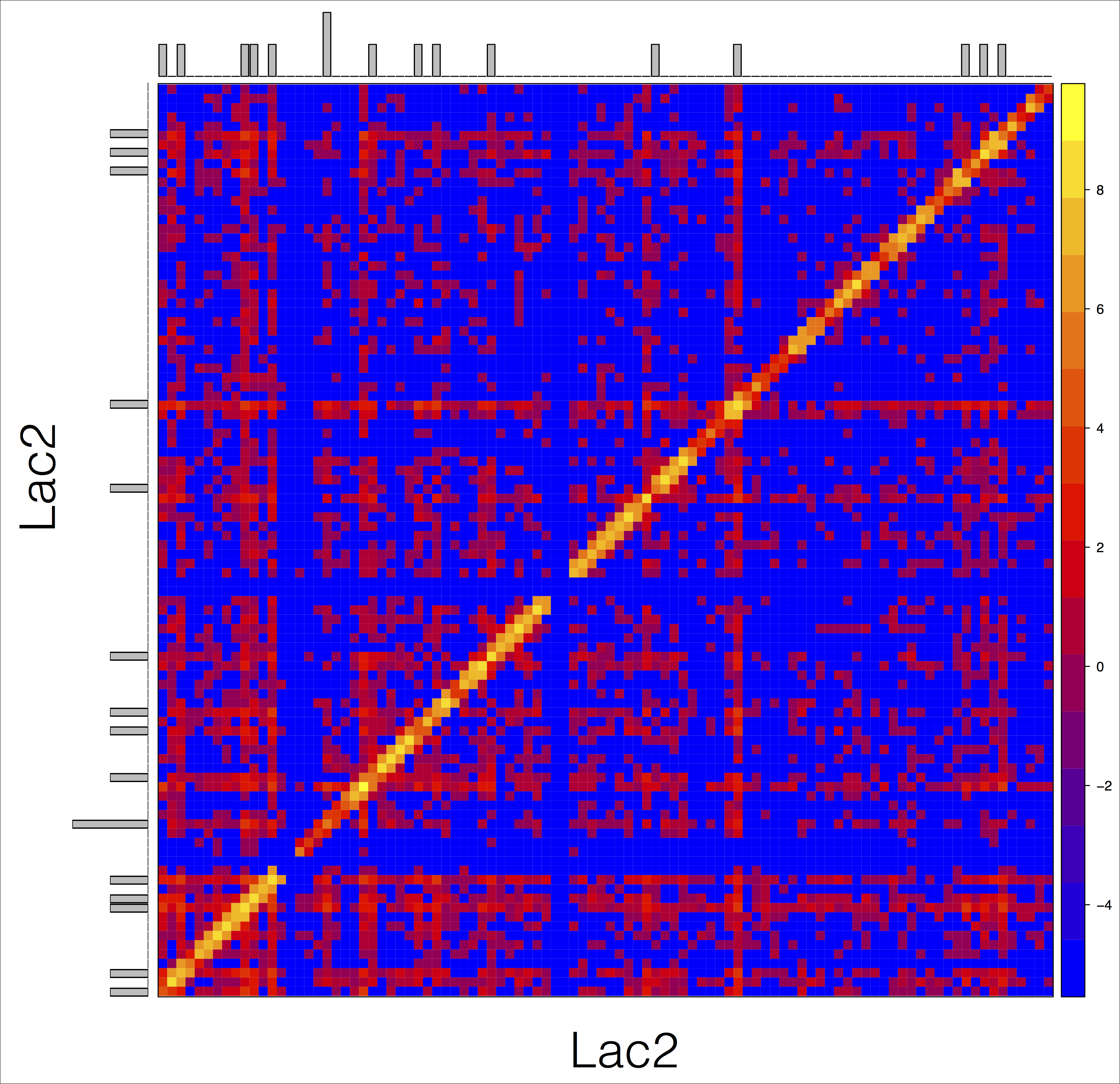

Supplement: Figure S5 — Contact maps show the number of Hi-C read pairs associating each region of the L. brevis plasmid 2. Contact values are Spearman rank correlation transformed following normalization. Pixels are sized to represent interactions between blocks sized at 1% of the interacting sequence. A signal indicative of circularity is not apparent. [file peerj-02-415-s005.png]

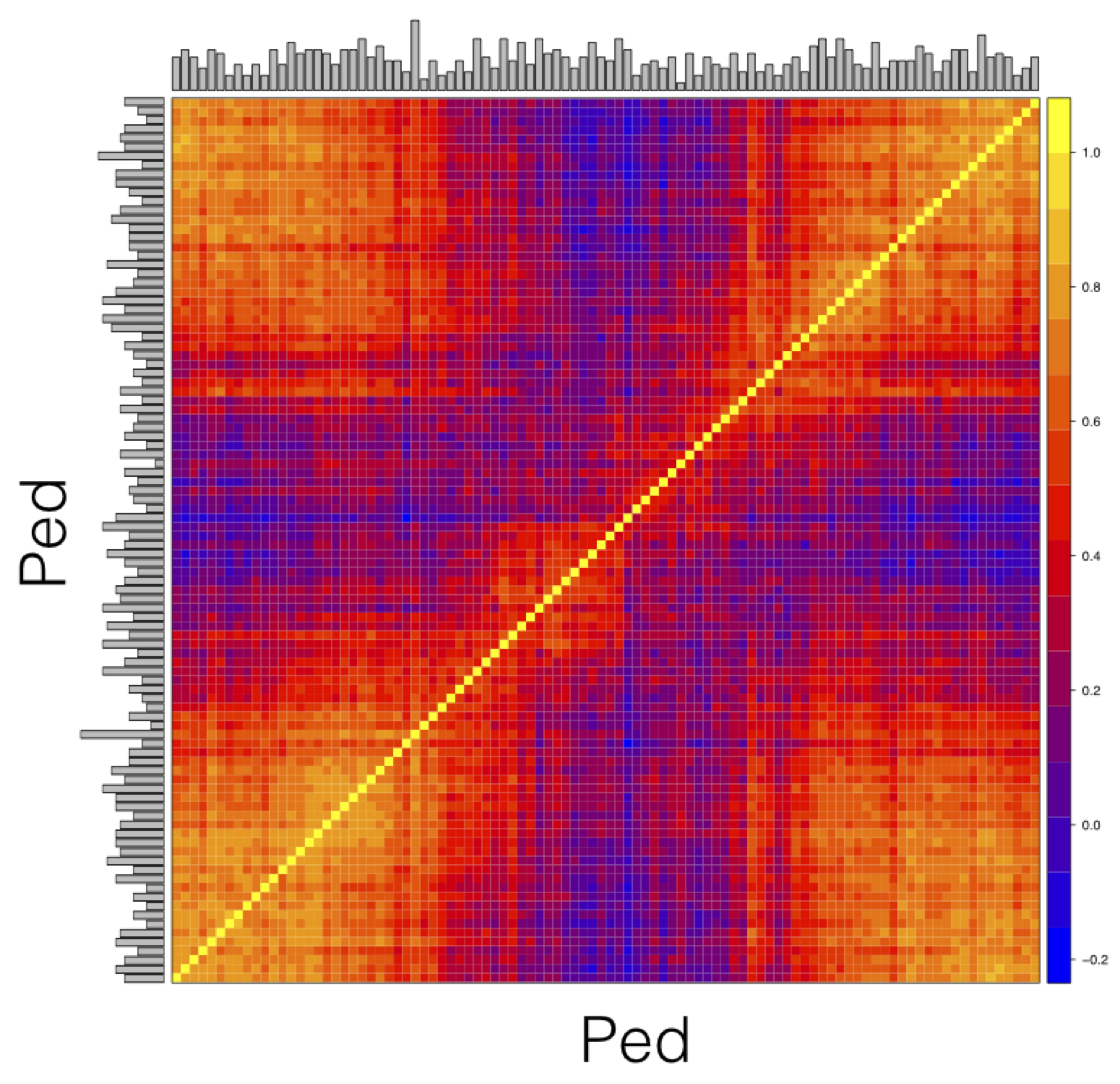

Supplement: Figure S6 — Contact frequency is visualized as a heat map, after normalization and application of the spearman rank correlation (matrix elements are the spearman correlation of the row and column of which they are the intersection). Circularity is apparent in the elevated contact between either end of the reference assembly sequence. [file peerj-02-415-s006.png]

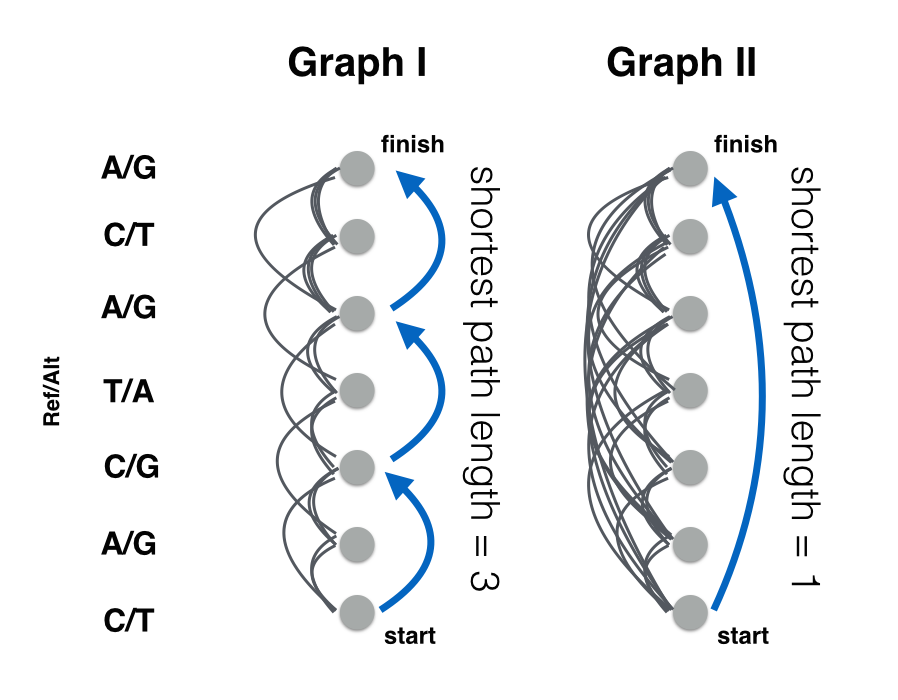

Supplement: Figure S7 — Two examples of variant graphs (non-data illustration). Variant nodes (circles) are linked by edges (light grey lines) derived from read pair data with small and medium (Graph I) or small, medium, and large (Graph 2) inserts. A path between two nodes (start, end) is illustrated and this path is shorter in the graph representing the dataset that includes larger-insert reads. [file peerj-02-415-s007.png]
