# Supplementary material for: Strain- and plasmid-level deconvolution of a synthetic metagenome by sequencing proximity ligation products
Source: PeerJ. 2014 May 27;2:e415. doi: 10.7717/peerj.415 (PMC4045339; doi:10.7717/peerj.415)
Supplement: Supplemental Information 1 [file peerj-02-415-s012.docx]

**Supplementary Methods: Hi-C of mixed bacterial cultures**

Hi-C was carried out by combining the cross-linking and cell wall digestion procedures described by (Umbarger et al., 2011) for bacterial 3-C experiments and the Hi-C protocol developed for mammalian cells (Lieberman-Aiden et al., 2009) with minor modifications:

1. Cross-Linking, Cell Wall Digestion, Lysis-part-I (according to Umbarger et al., 2011)
   1. 5 × 10^7^ Cells were transferred into a 50 mL centrifuge tube and washed three times in 25 ml of TE buffer (pH8.0) by centrifugation for 5 minutes at 4000 rpm at 4°C.
   2. Cells were resuspended at an OD600 of 0.2 in TE and 37% formaldehyde was added to a final concentration of 1%.
   3. Cells were incubated at room temperature for 30 min and subsequently for another 30 minutes on ice.
   4. The formaldehyde was quenched by adding glycine to a final concentration of 0.125M; mixed by inverting the tube, and incubated on ice for 10 minutes.
   5. After centrifugation cells were resuspended in 1mL TE.
   6. Fresh lysozyme (10mg/mL) was prepared and 1/10 vol. added to the cells; lysozyme digestion was carried out was incubated at room temperature for 15 minutes with occasional mixing via pipetting.
   7. Cells were pelleted by centrifugation at 4°C and were re-suspended in 1x TE to a concentration of 1.0 ×10^7^ cells/μL. Fresh lysozyme (10mg/mL) was prepared and 1/10 vol. added to the cells and mixed by inversion of tubes. The resulting mixture was incubated at room temperature for 15 minutes with occasional mixing via inversion.
   8. 10% SDS was added to a final concentration of 0.5% and was incubated with the lysed cells for 10 minutes.
2. Lysis-part-II, Restriction Digestion, Labeling & Ligation (modified from Lieberman-Aiden et al., 2009)
   1. Cells were pelleted by centrifugation at 4°C and resuspended in 550μL Hi-C lysis buffer (10mM Tris-HCl pH8.0, 10mM NaCl, 0.2% Igepal CA-630, 50μL protease inhibitors (Sigma, St. Louis, MO). Cells were incubated on ice for at least 15 minutes.
   2. The following treatments followed the Lieberman-Aiden et al. (2009) protocol exactly:
      1. Dounce homogenizer treatment (likely unnecessary).
      2. Washes in NEBuffer2.
      3. The cells were resuspended in a total volume of 100μL NEBuffer 2 and split in two 50μL aliquots.
   3. Each of the aliquots was treated exactly as described (Lieberman-Aiden et al., 2009):
      1. Incubation in the presence of 0.1% SDS at 65°C for 10 minutes.
      2. Quenching of SDS with Triton X-100.
      3. Restriction digestion overnight with *Hind*III.
   4. To ﬁll in and label the DNA ends, 1.5μL 10 mM dATP, 1.5μL 10 mM dGTP, 1.5μL 10 mM dTTP, 14μL 0.4 mM biotin-14-dCTP (Invitrogen, Carlsbad, CA) and 10μL 5U/pL Klenow (NEB) were added to the tubes. The reactions were incubated at 37°C for 45 minutes and then placed on ice.
   5. The following steps were executed for the two aliquots exactly as described by Lieberman-Aiden et al. (2009):
      1. Enzyme inactivation.
      2. Blunt–end ligation in a large volume (*.2 mL) at 16°C for 4 hours.
      3. Proteinase K digestion.
      4. Two large volume phenol/chloroform extractions.
      5. Ethanol precipitation.
      6. Two small volume phenol/chloroform extractions.
      7. Ethanol precipitation.
      8. RNAse A digestion.
      9. Pooling of the two aliquots.
   6. The human specific Hi-C QC PCR reactions in the Lieberman et al. (2009) protocol were omitted.
   7. The following steps were executed for the two aliquots exactly as described by Lieberman-Aiden et al. (2009):
      1. T4-polymerase end digestion for biotin removal.
      2. Phenol/chloroform extraction.
      3. Ethanol precipitation.
   8. The DNA was sheared to fragments sizes between 300 and 500 bp in aliquots of 140μL with a Covaris E220 instrument in Covaris microTUBES at a duty cycle of 10%, at intensity 4, Cycles/burst 200, for 55 seconds.
   9. The following steps were executed for the two aliquots exactly as described by Lieberman-Aiden et al. (2009):
      1. DNA end-repair.
      2. Gel electrophoresis.
      3. Size selection for fragment sizes between 280 and 420bp.
      4. Gel extraction.
   10. A-tailing and adapter ligation were carried out according to standard protocols (e.g. Henry et al., 2014).
   11. Biotin pull-down with 50μL MyOne Streptavin C1 Dynabeads (Invitrogen, Carlsbad, CA) was carried out according to the protocol of the manufacturer.
   12. The bead captured Hi-C library was split in two aliquots and amplified in two 50μL PCR reactions using KAPA Hifi MM polymerase reagents (Kapa Biosystems). The beads were resuspended thoroughly immediately before the start of the PCR.
   13. Reactions were finally cleaned up with Ampure XP beads (Agencourt) according to manufacturer protocols.
   14. The library was sequenced on an Illumina Miseq machine with 155 bp paired end reads.

References

Henry, Isabelle M., Ugrappa Nagalakshmi, Meric C. Lieberman, Kathie J. Ngo, Ksenia V. Krasileva, Hans Vasquez-Gross, Alina Akhunova et al. "Efficient Genome-Wide Detection and Cataloging of EMS-Induced Mutations Using Exome Capture and Next-Generation Sequencing." The Plant Cell Online (2014): tpc-113.

Lieberman-Aiden, E., Van Berkum, N. L., Williams, L., Imakaev, M., Ragoczy, T., Telling, A., Amit, I., et al. (2009). Comprehensive mapping of long-range interactions reveals folding principles of the human genome. *Science (New York, N.Y.)*, *326*(5950), 289–93. doi:10.1126/science.1181369

Umbarger, M. a, Toro, E., Wright, M. a, Porreca, G. J., Baù, D., Hong, S.-H., Fero, M. J., et al. (2011). The three-dimensional architecture of a bacterial genome and its alteration by genetic perturbation. *Molecular cell*, *44*(2), 252–64. doi:10.1016/j.molcel.2011.09.010
